# An Entropic Value-at-Risk-Inspired Load-Balancing Routing Algorithm for LEO Satellite Networks

**DOI:** 10.3390/s26206627

**Published:** 2026-10-19

**Authors:** Shuqi Wang, Huizi Guo, Weijie Deng

**Affiliations:** School of Communication and Information Engineering, Xi’an University of Science and Technology, Xi’an 710600, China; 24207223082@stu.xust.edu.cn (H.G.); 23207040041@stu.xust.edu.cn (W.D.)

**Keywords:** low Earth orbit (LEO) satellite networks, soft actor–critic (SAC), Entropic Value-at-Risk (EVaR), multi-agent reinforcement learning

## Abstract

In sixth-generation (6G) space–air–ground integrated networks, risk-neutral reinforcement-learning-based routing methods for low Earth orbit (LEO) satellite networks remain susceptible to severe tail latency. To address this issue, this paper proposes a novel Entropic Value-at-Risk (EVaR)-inspired routing algorithm with risk regularization for low-return actions. The routing process in an LEO satellite network is formulated as an event-driven partially observable Markov decision process. Within a multi-agent soft actor–critic framework, an EVaR-inspired state-level risk proxy for low-return actions is introduced and incorporated into the actor loss as a risk regularizer. This design enables the joint optimization of expected routing returns and the mitigation of low-return action risk. Simulation results obtained in a simplified LEO network using Iridium orbital parameters demonstrate that, compared with the multi-agent soft actor–critic algorithm, the proposed algorithm reduces the average end-to-end delay and the 99th-percentile end-to-end delay by 19.2% and 32.9%, respectively. It also reduces the average and maximum queueing delays by 79.4% and 57.0%, respectively, with approximately no packet loss observed during the simulations. These results demonstrate that the proposed algorithm enables efficient and reliable routing in LEO satellite networks under high-load conditions.

## 1. Introduction

Within the architecture of sixth-generation (6G) space–air–ground integrated communication networks, low Earth orbit (LEO) satellites serve as a key infrastructure component for enabling wide-area coverage and highly reliable ubiquitous access. By establishing seamless three-dimensional communication links with global reach [1], LEO satellites can provide stable, low-latency, and high-bandwidth connectivity for applications such as autonomous driving and emergency communications [2].

To improve the adaptability of routing schemes in LEO satellite networks to dynamic topology and traffic-load variations, Zhang et al. [3] proposed the RTLBR algorithm. The algorithm uses regional traffic-load information to calculate per-hop traffic-splitting ratios and employs a traffic-light-based strategy to dynamically adjust its parameters. It demonstrates significant advantages in reducing end-to-end delay and packet loss while improving link utilization. Ma et al. [4] proposed the ACLR-SR algorithm by integrating segment routing with ant colony optimization. Through an improved heuristic function and a negative-feedback mechanism for feeder links, the algorithm effectively mitigates link congestion, reduces packet loss, and maintains the continuity of backhaul services. Dun et al. [5] proposed the DLR algorithm, which exploits the predictable movement patterns of satellites to proactively prevent congestion, thereby reducing end-to-end delay and packet loss while improving traffic distribution. However, these methods rely on manually configured thresholds and fixed weighting parameters, limiting their ability to adapt in real time to rapidly changing network conditions. In addition, Wang et al. [6] investigated a hybrid routing algorithm based on service scheduling in software-defined satellite networks.

Owing to their strong adaptability, reinforcement learning (RL) methods have emerged as an important research direction for routing in LEO satellite networks. Among single-agent deep reinforcement learning approaches, Dong et al. [7] proposed the DQN-LLRA algorithm, which uses network-state information from neighboring nodes and obtains an optimal routing decision model through iterative training. Compared with the Q-learning-based scheme, DQN-LLRA reduces the maximum and average queue utilization along routing paths by 5% and 13%, respectively. For dynamic multilayer LEO optical satellite networks, Wei et al. [8] developed the DRL-SPP service-path prediction strategy, which integrates multi-objective optimization with deep reinforcement learning to enable predictive resource scheduling and load balancing. Compared with the TD3 and DDPG algorithms, DRL-SPP achieves faster convergence and greater training stability. To address dynamic multipath routing in LEO satellite networks with incomplete network-state information, Liang and Wang [9] proposed the SIDMRA algorithm. The algorithm exploits temporal correlations in network states to reconstruct missing information, uses DDPG to generate link weights, and combines these weights with the K-shortest-path algorithm to dynamically construct routing strategies. It effectively reduces end-to-end delay and packet loss and maintains stable performance under different proportions of missing network-state information.

Among multi-agent reinforcement learning approaches, Ran et al. [10] proposed the GraphPR algorithm for dynamic packet routing in LEO satellite networks. A graph attention network is employed to encode and exchange one-hop neighborhood information perceived by individual satellites, together with a residual shortest-path hop-count (RSPH) mechanism. The algorithm achieves lower average transmission delay and packet loss while maintaining higher network throughput. More recently, Rao et al. [11] proposed DGA-IES, which combines deep graph-attention-based topology perception with an incremental evolution strategy. The graph-attention module captures inter-satellite connectivity and node- and link-resource states, while the incremental evolutionary process improves routing reconvergence under rapidly changing topologies. DGA-IES primarily addresses topology representation and routing reconvergence, whereas the present work focuses on event-driven decentralized routing and congestion-related tail-risk mitigation. For multibeam LEO satellite networks, Lee et al. [12] proposed a multi-agent deep Q-network (MADQN)-based handover strategy. By jointly considering quality-of-service constraints, load balancing, and the differences in handover costs between intra-satellite and inter-satellite beam handovers, the strategy jointly minimizes the number of handovers and maximizes network throughput. Liu et al. [13] developed the DRL-MPCR cooperative multipath routing scheme for ultra-dense LEO satellite networks. The scheme employs a water-filling-based traffic scheduling strategy to dynamically allocate traffic flows and minimize the maximum path cost in cooperative multipath transmission. It incurs lower routing-signaling overhead and delivers improved cooperative multipath transmission performance in terms of transmission delay, packet loss, and link-load balancing.

However, most existing reinforcement learning-based routing algorithms primarily optimize risk-neutral objectives, such as average end-to-end delay and packet loss. In delay-sensitive applications, including real-time voice services and emergency communications, local congestion or sudden queue buildup may lead to severe tail latency. To address this issue, this paper proposes an Entropic Value-at-Risk (EVaR)-inspired routing algorithm with risk regularization for low-return actions. By suppressing risky low-return routing actions, the proposed algorithm aims to improve decision-making robustness under congested network conditions and reduce both average end-to-end delay and tail latency. The main contributions of this paper are summarized as follows:

(1) A distributed decision-making model for event-driven routing is established by formulating the hop-by-hop packet forwarding process as an event-driven partially observable Markov decision process (POMDP). This model enables each satellite to make autonomous routing decisions in real time based on locally observed information and the states of neighboring satellites.
(2) A novel multi-agent soft actor–critic (MASAC) routing algorithm, termed MASAC-EVaR, is proposed with EVaR-inspired risk regularization for low-return actions. Within the MASAC framework, an EVaR-inspired state-level surrogate term for low-return action risk is introduced and incorporated into the actor loss as a risk regularizer, imposing exponentially weighted penalties on inferior actions in the policy distribution. By leveraging local observations that include queueing delay, link capacity, and node load, the proposed algorithm jointly improves end-to-end delay, load balancing, and packet delivery reliability.
(3) The incremental effect of the proposed risk regularizer is evaluated against the unchanged MASAC backbone and the risk-aware PRIMAL-CVaR baseline using mean, 99th-percentile (P99), and conditional value-at-risk (CVaR) delay metrics under different traffic loads.

## 2. System Model

**Figure 1:**
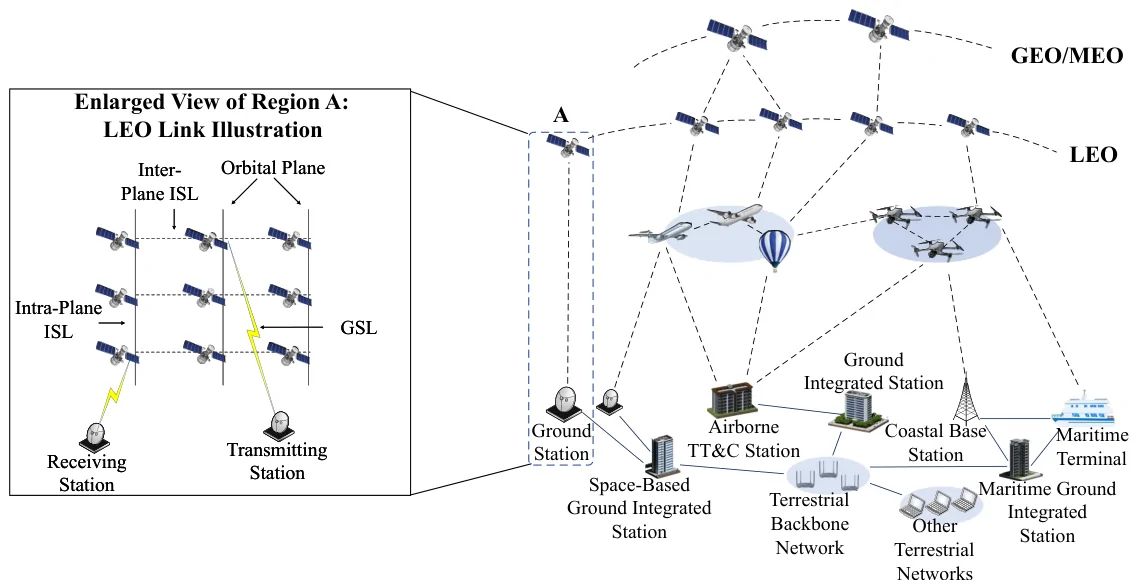
Schematic of the integrated space–air–ground network and low Earth orbit (LEO) links. Black dashed lines schematically represent wireless communication links, while the blue dashed rectangle indicates Region A, which is enlarged in the inset.

A simplified LEO satellite network based on the orbital parameters of the Iridium constellation is considered for routing evaluation. The satellite network is modeled as an undirected graph G=(V,E) [14], where *V* denotes the set of satellite nodes and *E* denotes the set of inter-satellite links (ISLs). The total number of satellites is given by N=P×S, where *P* denotes the number of orbital planes and *S* denotes the number of satellites in each orbital plane [15]. For the Iridium constellation, P=6 and S=11. The inter-satellite links (ISLs) and ground–satellite links (GSLs) are illustrated in Figure 1.

The network adopts a simplified four-neighbor topology based on the orbital parameters of the Iridium constellation. Candidate ISLs connect adjacent satellites within each orbital plane and index-matched satellites in neighboring planes, including the wraparound connection between the first and last planes. Link availability is determined by geometric visibility. Iridium-specific polar-region link deactivation and cross-seam link exclusion are not explicitly modeled. The action mask excludes links marked as unavailable by the simulated topology.

### 2.1. Link Capacity Model

The simulation adopts a fixed-capacity communication model to focus on routing and queueing performance. For an available ground–satellite link (GSL), the data rate is

(1) $$R_{ij}^{\mathrm{GSL}}\left(t\right)=R_{\mathrm{GSL}}=1.0\;\mathrm{Gbps}.$$

For an available inter-satellite link (ISL), the data rate is

(2) $$R_{ij}^{\mathrm{ISL}}\left(t\right)=R_{\mathrm{ISL}}=0.2\;\mathrm{Gbps}.$$

The availability of a candidate ISL is determined by geometric visibility:

(3) $$\chi_{ij}\left(t\right)=\left\{\begin{array}{cc} 1, & d_{ij}\left(t\right)\leq d_{\max},\\ 0, & d_{ij}\left(t\right)>d_{\max}, \end{array}\right.$$

where $d_{ij}\left(t\right)$ denotes the distance between satellites *i* and *j*, and $d_{\max}$ is the maximum geometric visibility distance. Link distance affects connectivity and propagation delay, but does not change the transmission rate of an active link. The simulation therefore represents a routing-level fixed-capacity abstraction, rather than a detailed optical or RF physical-layer model.

### 2.2. Communication Delay Models

The single-hop delay incurred by packet *p* over link (i,j) at the *h*-th hop depends on its arrival time, $\tau_{p}^{h}$, at the transmitting node of that hop. If packet *p* arrives at the transmitting node of link (i,j) at time $t=\tau_{p}^{h}$, its single-hop delay, $D_{p,ij}^{h}$, can be decomposed into three components: propagation delay, transmission delay, and queueing delay [16]:

(4) $$D_{p,ij}^{h}=D_{ij}^{P}\left(\tau_{p}^{h}\right)+D_{ij}^{T}\left(L_{p},\tau_{p}^{h}\right)+D_{ij}^{Q}\left(\tau_{p}^{h}\right).$$

The propagation delay $D_{ij}^{P}\left(t\right)$ represents the time required for a signal to propagate from node *i* to node *j* over link (i,j) at time *t*. It is determined by the Euclidean distance $d_{ij}\left(t\right)$ between the two nodes and the speed of light $\kappa_{c}$, and is given by

(5) $$D_{ij}^{P}\left(t\right)=\frac{d_{ij}\left(t\right)}{\kappa_{c}}.$$

The transmission delay $D_{ij}^{T}\left(L_{p},t\right)$ is the time required to push all $L_{p}$ bits of packet *p* onto communication link (i,j) at time *t*, and is given by

(6) $$D_{ij}^{T}\left(L_{p},t\right)=\frac{L_{p}}{R_{ij}\left(t\right)}.$$

where $R_{ij}\left(t\right)$ corresponds to $R_{ij}^{\mathrm{GSL}}\left(t\right)$ for a ground–satellite link or $R_{ij}^{\mathrm{ISL}}\left(t\right)$ for an inter-satellite link.

The queueing delay $D_{ij}^{Q}\left(t\right)$ is the time a packet spends waiting in the output buffer before transmission begins. Under a first-in, first-out (FIFO) queueing discipline, this delay is equal to the sum of the transmission delays of all packets ahead of it in the output queue associated with the same outgoing link. If packet *p* arrives at node *i* at time *t* and is forwarded to next-hop node *j*, its queueing delay can be approximated by

(7) $$D_{ij}^{Q}\left(t\right)=\sum_{q\in P_{ij}^{Q}\left(t\right)}\frac{L_{q}}{R_{ij}\left(t\right)}.$$

Queueing delay is a direct indicator of local congestion, as congestion at a node typically causes its packet queue to grow [17]. Therefore, the cumulative queueing delay experienced by a packet during end-to-end transmission reflects the level of congestion encountered along its routing path and serves as a key metric for evaluating load-balancing and congestion-avoidance performance.

## 3. Load-Balancing-Based MASAC Routing Algorithm

Given the dynamic variations in LEO satellite network topology and traffic load, routing policies must strike a balance among effective exploration, training stability, and sample efficiency. Soft actor-critic (SAC) incorporates a maximum-entropy objective to prevent premature policy convergence. Moreover, its off-policy learning framework and twin Q-networks improve sample efficiency and training stability while alleviating value overestimation.

Routing decisions in LEO satellite networks are inherently event-driven. Packet-forwarding decisions are triggered when packets arrive at satellite nodes, using the most recently updated network state and action mask. Accordingly, the LEO satellite network routing problem is formulated in this paper as an event-driven partially observable Markov decision process (POMDP).

During multi-agent training, all satellite agents share the parameters of the actor and critic networks. The interaction experiences collected by different agents are stored in a shared experience replay buffer, denoted by D. Each transition stored in the replay buffer generally consists of the current state, the selected action, the received reward, the next state, and a termination indicator. The shared actor–critic parameters and replay buffer are standard components of the parameter-sharing MASAC backbone rather than methodological contributions of this work. Compared with independent-agent implementations, this design reduces model complexity and improves experience reuse among homogeneous satellites while preserving decentralized execution based on local and neighboring states. MASAC-EVaR retains this backbone unchanged; its incremental contribution over MASAC is the EVaR-inspired regularization term introduced into the actor objective. The state space, action space, reward function, and single-hop delay cost metric are defined as follows:

**(1) State Space:** The global state comprises information about the satellite network topology, inter-satellite link connectivity, node buffer occupancy, queue states, and packet states. Because an individual agent cannot fully access the global state when making routing decisions, the routing process is modeled as a partially observable process. At decision time *t*, the agent obtains a local observation $o_{t}\in O$, from which an input representation is extracted as $z_{t}=f\left(o_{t}\right)\in Z$. This representation is then fed into both the policy and critic networks.

The state vector adopted in this study is 94-dimensional. It primarily contains information on the current node load, the locations of candidate next-hop satellites in each neighboring direction, relative distances, progress toward the destination, queueing delays, transmission delays, residual link capacities, and the load conditions of neighboring nodes. Based on this state representation, the agent can comprehensively evaluate the available routing options and determine the forwarding direction of the current packet using only locally observable information.

**(2) Action Space:** The action space is defined as the following discrete set:

(8) $$A=\{a_{E},a_{S},a_{W},a_{N}\}.$$

where the four actions correspond to forwarding the packet through the eastbound, southbound, westbound, and northbound inter-satellite links, respectively. Therefore, the cardinality of the action space is |A|=4.

**(3) Reward Function:** The reward function is designed to encourage the agent to improve the packet delivery ratio and forwarding progress, and serves as the explicit optimization objective during training.

For a normal packet-forwarding event, the immediate reward is defined as

(9) $$r_{t}=\Delta p_{t}+I_{\mathrm{SUCC}}\left(1+k_{s}b_{t}\right).$$

where $\Delta p_{t}$ denotes the normalized distance-progress reward resulting from the current forwarding action; $I_{\mathrm{SUCC}}$ is an indicator of successful delivery, taking a value of 1 if the packet successfully reaches the destination ground station and 0 otherwise; $b_{t}$ denotes the packet size; and $k_{s}$ is the reward coefficient for successful delivery.

When a packet-drop event occurs due to TTL expiration, buffer overflow, or the absence of an available next hop, the immediate reward is replaced by a packet-drop penalty:

(10) $$r_{t}=r_{\mathrm{drop}},\;r_{\mathrm{drop}}<0.$$

The packet-drop penalty is introduced to discourage ineffective forwarding actions and the selection of routes with a high risk of delivery failure.

**(4) Single-Hop Delay Cost Metric:** The single-hop delay cost metric characterizes the delay incurred at each hop and is defined as the total time required to complete the current hop:

(11) $$C_{t}=D_{p,ij}^{h}.$$

where $D_{p,ij}^{h}$ denotes the single-hop delay, comprising propagation delay, transmission delay, and queueing delay. This metric is used for end-to-end delay accumulation, delay-component analysis, and simulation statistics, but does not directly enter the loss functions of either the policy network or the critic network. Instead, the queueing delay, transmission delay, residual link capacity, and node load associated with each candidate forwarding direction are incorporated into the state representation, thereby indirectly affecting action-value estimation and next-hop selection.

### Discrete-Action MASAC Algorithm

Soft Actor-Critic (SAC) [18] is an off-policy actor–critic algorithm developed under the maximum-entropy reinforcement learning framework. Its central idea is to augment the objective of maximizing the expected return with a policy-entropy term, thereby encouraging sustained exploration. For the discrete-action routing problem considered in this paper, the maximum-entropy objective of SAC can be formulated as

(12) $$J\left(\pi_{\theta }\right)=E_{\pi_{\theta }}\;\left[\sum_{t=0}^{\infty }\gamma^{t}\left(r_{t}+\alpha H\left(\pi_{\theta }\left(\cdot |z_{t}\right)\right)\right)\right].$$

The policy entropy is defined as

(13) $$H\left(\pi_{\theta }\left(\cdot |z_{t}\right)\right)=-\sum_{a\in A_{v}\left(z_{t}\right)}\pi_{\theta }\left(a|z_{t}\right)\log\pi_{\theta }\left(a|z_{t}\right).$$

where α>0 is the temperature parameter that controls the relative weight of the entropy term, and $H(\pi_{\theta }\left(\cdot |z_{t}\right))$ denotes the entropy of the policy distribution over the valid action set $A_{v}\left(z_{t}\right)$, conditioned on $z_{t}$.

To mitigate Q-value overestimation, SAC typically employs twin Q-networks. In this study, the clipped double-Q mechanism is adopted, whereby the smaller of the two Q-value estimates is used, as shown in (14).

(14) $${\bar{Q}}_{R}\left(z_{t},a\right)=\min_{i=1,2}Q_{R,i}\left(z_{t},a;\omega_{i}\right).$$

The target Q-value for the return critic is defined as

(15) $$y_{t}^{R}=r_{t}+\gamma \left(1-d_{t}\right)\sum_{a^{\prime }\in A_{v}\left(z_{t+1}\right)}\pi_{\theta }\left(a^{\prime }|z_{t+1}\right)\left[{\bar{Q}}_{R}\left(z_{t+1},a^{\prime }\right)-\alpha \log\pi_{\theta }\left(a^{\prime }|z_{t+1}\right)\right].$$

The two critic networks are updated by minimizing the mean-squared temporal-difference (TD) error defined in (16). The total loss of the twin critics is given by $L_{Q_{R}}=L_{Q_{R,1}}+L_{Q_{R,2}}$.

(16) $$L_{Q_{R,i}}\left(\omega_{i}\right)=E_{D}\;\left[{\left(Q_{R,i}\left(z_{t},a_{t};\omega_{i}\right)-y_{t}^{R}\right)}^{2}\right],\;i=1,2.$$

For a discrete action space, the loss function of the actor network is defined in (17). The entropy regularization term $\alpha \log\pi_{\theta }\left(a|z_{t}\right)$ encourages continued policy exploration, while $-{\bar{Q}}_{R}\left(z_{t},a\right)$ represents the negative of the conservative return estimate. By minimizing the actor loss, the policy increases the probabilities assigned to actions with higher expected returns.

(17) $$L_{\pi }^{\mathrm{SAC}}\left(\theta \right)=E_{z_{t}∼D}\;\left[\sum_{a\in A_{v}\left(z_{t}\right)}\pi_{\theta }\left(a|z_{t}\right)\left(\alpha \log\pi_{\theta }\left(a|z_{t}\right)-{\bar{Q}}_{R}\left(z_{t},a\right)\right)\right].$$

## 4. An EVaR-Inspired Risk-Regularized Routing Algorithm for Low-Return Actions

When the network encounters localized congestion, abrupt surges in buffer occupancy, or rapid changes in link conditions, SAC, which optimizes only the expected return, may still assign non-negligible probabilities to low-return actions. Such actions can induce severe performance degradation, including excessively long routing detours and persistent queueing, thereby increasing the upper tail of the end-to-end delay distribution. To address this issue, we introduce, without modifying the SAC network architecture, an EVaR-inspired state-level risk proxy for low-return actions. By regularizing the probability mass that the current policy assigns to such actions, the proposed method jointly optimizes the expected return while suppressing the risk associated with low-return actions.

### 4.1. Entropic Value-at-Risk Theory and an EVaR-Inspired State-Level Risk Proxy for Low-Return Actions

Entropic Value-at-Risk (EVaR) is a tail-risk measure defined in terms of the moment-generating function [19]. Its standard form is expressed as

(18) $${\mathrm{EVaR}}_{1-\delta }\left(X\right)=\underset{\eta >0}{\inf}\left\{\frac{1}{\eta }\left[\log E\;\left(e^{\eta X}\right)-\log\delta \right]\right\}.$$

Here, *X* denotes the loss random variable, η>0 is the auxiliary optimization variable, and δ∈(0,1) is the tail-probability level, corresponding to a confidence level of 1−δ.

In routing tasks, directly defining *X* as the cumulative delay cost over an entire trajectory would require sampling from the trajectory distribution induced by the policy and estimating the corresponding exponential moment. This procedure is computationally expensive and difficult to implement reliably at every policy update. Therefore, rather than directly evaluating the standard trajectory-level EVaR, we fix the risk-sensitivity parameter η and construct a state-level risk proxy for low-return actions inspired by its exponential-moment formulation.

The exponential-moment structure of EVaR was adopted as the conceptual basis for constructing a smooth and differentiable regularization term using the existing return critics. The resulting formulation operates on the state-wise distribution of estimated action values under the current policy, rather than on a trajectory-level delay-loss distribution. Therefore, it is regarded as an EVaR-inspired entropic regularizer, without claiming equivalence to standard EVaR or direct optimization of delay-tail risk.

The risk proxy is constructed using the return critic $Q_{R}$. For all candidate actions available at state $z_{t}$, the conservative return estimate obtained by aggregating the twin Q-functions is defined as

(19) $${\bar{Q}}_{R}\left(z_{t},a\right)=\min_{i=1,2}Q_{R,i}\left(z_{t},a;\omega_{i}\right).$$

In routing tasks, a higher ${\bar{Q}}_{R}$ indicates that an action is associated with greater forwarding progress, a higher probability of successful delivery, or a lower packet-loss risk, whereas a lower ${\bar{Q}}_{R}$ reflects a lower long-term aggregate return. Localized congestion, routing detours, and elevated packet-loss risk typically reduce an action’s value. Accordingly, we define the probability mass assigned by the policy to low-return actions as a state-level risk proxy for low-return actions. Using the action-value estimates provided by the return critic, this proxy regularizes the policy’s exposure to low-return actions at each decision step.

Based on the estimated returns of the candidate actions available at state $z_{t}$, the state-level risk proxy for low-return actions is defined as follows:

(20) $$\rho_{\eta }\left(z_{t};\theta \right)=\frac{1}{\eta }\log\;\left[\sum_{a\in A_{v}\left(z_{t}\right)}\pi_{\theta }\left(a|z_{t}\right)\exp\;\left(-\eta {\bar{Q}}_{R}\left(z_{t},a\right)\right)\right].$$

Equation (20) is inspired by the exponential-moment structure of EVaR but is not the standard EVaR of a trajectory-level loss distribution. Unlike standard EVaR, which involves a confidence level and optimization over the exponential parameter, the proposed formulation applies a fixed risk-sensitivity parameter η>0 to the state-wise action-value distribution. It is therefore interpreted as an EVaR-inspired entropic risk proxy for low-return actions.

The proxy exponentially weights the negated return estimates of feasible actions, thereby assigning larger risk weights to actions with lower estimated returns. The coefficient β controls the strength of risk regularization; when β=0, the actor loss reduces to that of the standard discrete SAC. Moreover, because the proxy explicitly depends on the action probability $\pi_{\theta }\left(a|z_{t}\right)$, its gradient can be backpropagated directly through the policy network without introducing an additional risk critic. This enables the policy to explicitly reduce the probability mass assigned to low-return actions.

The proposed risk proxy is constructed from the surrogate action loss $-{\bar{Q}}_{R}\left(z_{t},a\right)$, rather than from the probability distribution of realized packet delays. As defined in Equation (9), the immediate reward incorporates forwarding progress and successful packet delivery, but does not explicitly include a delay penalty. Although queueing delay, residual link capacity, and node load are included in the state representation, their influence on policy decisions is indirect.

Consequently, the regularizer should be interpreted as acting on estimated action returns rather than directly controlling delay-tail risk. The reductions in queueing delay and end-to-end tail latency observed in the simulations are empirical performance outcomes.

### 4.2. MASAC-EVaR-Based Routing Algorithm with Risk Regularization for Low-Return Actions

**Figure 2:**
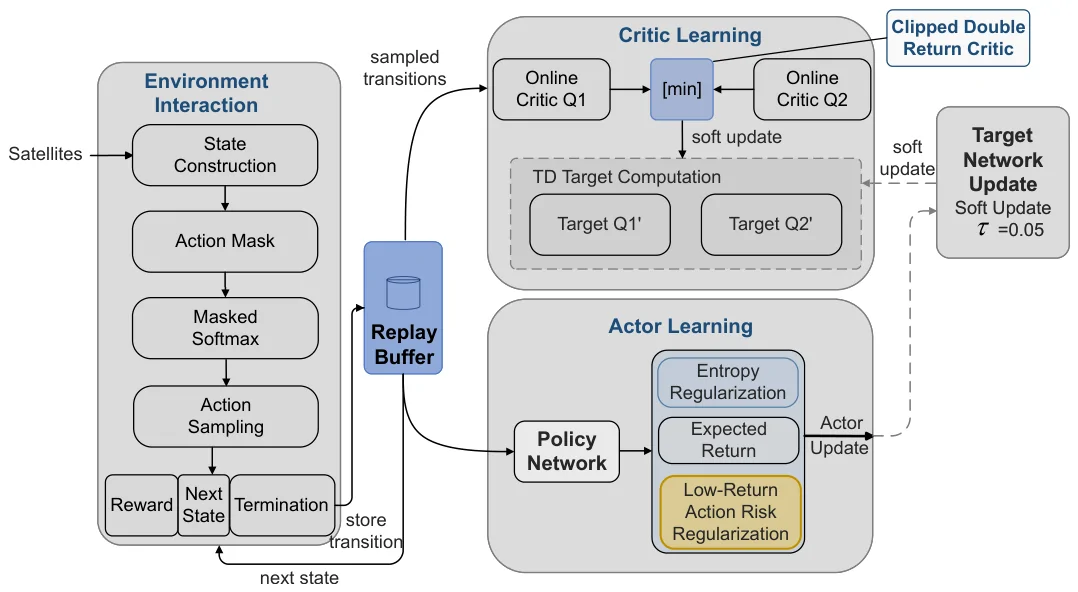
Framework of the multi-agent soft actor–critic (MASAC) algorithm with Entropic Value-at-Risk (EVaR)-inspired regularization (MASAC-EVaR).

Built upon the standard SAC framework, MASAC-EVaR introduces an EVaR-inspired state-level risk proxy for low-return actions, constructed from the Q-value estimates of the return critics. The overall algorithmic framework is illustrated in Figure 2. The two return critics, $Q_{R,1}$ and $Q_{R,2}$, independently estimate the expected cumulative discounted return for each state–action pair, and their outputs are aggregated according to Equation (19) to obtain a conservative return estimate.

The critic networks are updated following the same procedure as in standard discrete SAC. Given a sampled minibatch, the target value is first computed as follows:

(21) $$y_{t}^{R}=r_{t}+\gamma \left(1-d_{t}\right)\sum_{a^{\prime }\in A_{v}\left(z_{t+1}\right)}\pi_{\theta }\left(a^{\prime }|z_{t+1}\right)\left[{\bar{Q}}_{R}\left(z_{t+1},a^{\prime }\right)-\alpha \log\pi_{\theta }\left(a^{\prime }|z_{t+1}\right)\right].$$

The critic networks are then updated by minimizing the following loss function:

(22) $$L_{Q_{R}}\left(\omega_{1},\omega_{2}\right)=\sum_{i=1}^{2}E_{D}\;\left[{\left(Q_{R,i}\left(z_{t},a_{t};\omega_{i}\right)-y_{t}^{R}\right)}^{2}\right].$$

MASAC-EVaR augments the standard SAC actor loss with a risk regularization term for low-return actions. The resulting actor loss is defined as follows:

(23) $$L_{\pi }^{\mathrm{EVaR}}\left(\theta \right)=E_{z_{t}∼D}\;\left[\sum_{a\in A_{v}\left(z_{t}\right)}\pi_{\theta }\left(a|z_{t}\right)\left(\alpha \log\pi_{\theta }\left(a|z_{t}\right)-{\bar{Q}}_{R}\left(z_{t},a\right)\right)+\beta \rho_{\eta }\left(z_{t};\theta \right)\right].$$

Here, β≥0 denotes the risk-regularization weight, which controls the contribution of the risk proxy to the actor update, and $\rho_{\eta }\left(z_{t};\theta \right)$ denotes the state-level risk proxy for low-return actions defined in Equation (20) of Section 4.1.

The proposed regularizer modifies the standard SAC actor objective through the entropic transformation of state-wise action values. For sufficiently small η, a cumulant expansion of Equation (20) gives

$$\rho_{\eta }\left(z_{t};\theta \right)=-E_{a∼\pi_{\theta }}\left[{\bar{Q}}_{R}\left(z_{t},a\right)\right]+\frac{\eta }{2}{\mathrm{Var}}_{a∼\pi_{\theta }}\left[{\bar{Q}}_{R}\left(z_{t},a\right)\right]+O\left(\eta^{2}\right).$$

Accordingly, substituting this approximation into Equation (23) shows that the additional regularization term both increases the effective weight of the expected Q-value component from 1 to 1+β and introduces a penalty on the dispersion of action values under the current policy.

Thus, the regularizer may influence action preferences and the exploitation–exploration balance, even with automatic entropy-temperature tuning. This approximation characterizes the small-η behavior and does not establish an exact equivalence at the fixed parameter value used in the experiments.

The observed delay improvements are evaluated empirically in Section 5. The present results do not isolate the effect of the entropic regularizer from possible changes in expected-Q weighting or policy entropy.

The temperature parameter α is updated using an automatic entropy-tuning mechanism to match the target entropy $H_{\mathrm{tar}}$. Its loss function is defined as follows:

(24) $$L_{\alpha }\left(\alpha \right)=E_{z_{t}∼D}\;\left[\sum_{a\in A_{v}\left(z_{t}\right)}\pi_{\theta }\left(a|z_{t}\right)\left(-\alpha \left(\log\pi_{\theta }\left(a|z_{t}\right)+H_{\mathrm{tar}}\right)\right)\right].$$

The target networks are updated using the following soft-update rule:

(25) $${\bar{\omega }}_{i}\leftarrow \tau \omega_{i}+\left(1-\tau \right){\bar{\omega }}_{i},\;i=1,2.$$

Here, τ∈(0,1) denotes the soft-update coefficient for the target critic networks.

It should be noted that the learning objective is shaped by the immediate reward defined in Equation (9) and the packet-drop penalty in Equation (10). These terms encourage forwarding progress and successful packet delivery while penalizing packet loss. Accordingly, the critic networks primarily evaluate how each action contributes to long-term forwarding efficiency and delivery reliability. Propagation delay, transmission delay, queueing delay, and node load are incorporated into the state representation for Q-value estimation and thereby influence policy decisions indirectly through the estimated action values. Consequently, the packet-delivery ratio is directly associated with the training return, whereas improvements in latency and load balancing primarily arise from the policy’s ability to perceive and respond to changes in the network state.

MASAC-EVaR therefore establishes a joint learning mechanism that combines expected routing-return optimization with risk suppression for low-return actions. Specifically, the critic networks estimate the long-term cumulative return of each routing action, the actor network generates the next-hop forwarding policy, and the risk-regularization term for low-return actions is incorporated into the actor loss to explicitly constrain the probability mass assigned to such actions.

The procedure of the MASAC-EVaR algorithm is summarized in Algorithm 1.


**Algorithm 1.** MASAC-EVaR algorithm1**Initialization:** policy-network parameters θ; twin return-critic parameters $\omega_{1}$ and $\omega_{2}$; target-critic parameters ${\bar{\omega }}_{1}\leftarrow \omega_{1}$ and ${\bar{\omega }}_{2}\leftarrow \omega_{2}$; temperature parameter α; experience replay buffer D.2**Input:** discount factor γ; risk-sensitivity coefficient η; risk-regularization weight β; soft-update coefficient τ; batch size *B*; replay buffer size; learning rate.3**Output:** trained policy-network parameters θ and critic-network parameters $\omega_{1},\omega_{2}$.4**for** e=1,2,…,E **do**5 Obtain the initial local observation $o_{0}$, encode it as the network input state $z_{0}=f\left(o_{0}\right)$, and retrieve the corresponding action mask $M_{0}$.6 **for**
 t=0,1,2,…,T−1
**do**7  Construct the valid action set $A_{v}\left(z_{t}\right)$ using the action mask $M_{t}$.8  Obtain the probability distribution over valid actions from the policy network and sample $a_{t}∼\pi_{\theta }\left(\cdot |z_{t},M_{t}\right)$.9  Execute $a_{t}$ to obtain $r_{t}$, $o_{t+1}$, $M_{t+1}$, and $d_{t}$; encode the next observation as $z_{t+1}=f\left(o_{t+1}\right)$ and store $\left(z_{t},M_{t},a_{t},r_{t},z_{t+1},M_{t+1},d_{t}\right)$ in D.10  Randomly sample a minibatch of *B* transitions from the experience replay buffer D.11  Compute the TD target $y_{t}^{R}$ using the target critic networks and the set of valid next-step actions.12  Minimize $L_{Q_{R}}\left(\omega_{1},\omega_{2}\right)$ to update the twin return-critic networks.13  Compute the low-return risk proxy $\rho_{\eta }\left(z_{t};\theta \right)$ based on the current policy distribution and conservative return estimates.14  Minimize $L_{\pi }^{\mathrm{EVaR}}\left(\theta \right)$ to update the policy network.15  Minimize $L_{\alpha }\left(\alpha \right)$ to update the entropy temperature parameter α.16  Soft-update the target critic networks: ${\bar{\omega }}_{i}\leftarrow \tau \omega_{i}+\left(1-\tau \right){\bar{\omega }}_{i}$, i=1,2.17  Set $z_{t}\leftarrow z_{t+1}$ and $M_{t}\leftarrow M_{t+1}$.18  **if**
$d_{t}=1$
**then break**19 **end**20
**end**



## 5. Simulation Results and Analysis

### 5.1. Simulation Settings

The satellite network comprises 66 satellites operating at an orbital altitude of 780 km and an inclination of $86.4^{\circ }$, together with three ground stations. Packet arrivals follow a Poisson process. Each packet carries a time-to-live (TTL) field initialized to 64, which is decremented by one at each satellite hop. The network-wide packet arrival rate was set to λ=15 packets/ms as the nominal traffic setting. Packets of 16 kbit and 64 kbit were generated with probabilities of 20% and 80%, respectively, resulting in an expected offered load of 816 Mbps. Additional traffic rates were evaluated to examine load-dependent performance.

**Figure 3:**
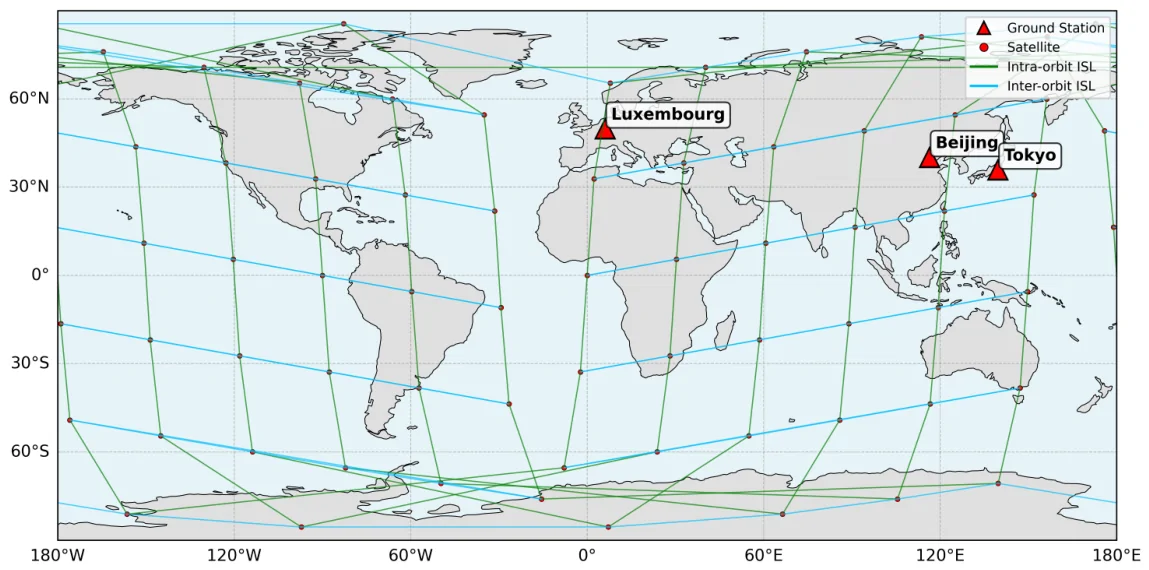
Simplified low Earth orbit (LEO) network topology based on Iridium constellation parameters.

The satellite network topology is illustrated in Figure 3, and the simulation parameters are summarized in Table 1.

The three ground stations are located in Beijing ($39.9087^{\circ }$ N, $116.3975^{\circ }$ E), Luxembourg ($49.6116^{\circ }$ N, $6.1319^{\circ }$ E), and Tokyo ($35.6762^{\circ }$ N, $139.6503^{\circ }$ E). For each generated packet, the source station is selected uniformly, and the destination is selected uniformly from the other two stations. Thus, the six directed source–destination pairs have equal expected traffic shares, with an arrival rate of λ/6 for each pair and zero self-traffic.

Satellite positions, link connectivity, and propagation delays were updated every 100 ms. In contrast, routing decisions were triggered by packet arrival events at individual satellites. Each forwarding decision used the most recent network state and action mask. Thus, the 100 ms interval represents periodic topology updates rather than the interval between routing decisions.

The actor and each of the twin return critics were implemented as fully connected neural networks with two hidden layers of 256 units each. Each hidden layer used layer normalization followed by a ReLU activation. The actor produced a categorical distribution over the valid next-hop actions, while each critic estimated the action values for all candidate actions. All linear layers were orthogonally initialized and optimized using Adam with a learning rate of $10^{-4}$. Gradient clipping with a maximum norm of 0.5 was applied during training.

Regarding link capacities, the GSL and ISL data rates were set to 1.0 Gbps and 0.2 Gbps, respectively. The buffer capacity of each node and link was set to 16 Mb to capture realistic congestion and queueing behavior. Each simulation lasted 30 s. Each learning-based routing method was trained once, and the resulting policy was evaluated over five runs with different evaluation random seeds. These seeds were applied to the evaluation stage rather than to independent policy retraining. Therefore, the five runs characterize performance variability under different stochastic evaluation conditions for a fixed trained policy. End-to-end throughput was calculated as the total number of successfully delivered bits at the destination ground stations across the five evaluation runs, divided by their total simulation duration of 150 s. Each successfully delivered packet was counted only once, regardless of the number of satellite hops traversed.

**Statistical analysis.** For the comparison between MASAC and MASAC-EVaR, the same five evaluation random seeds were used for both fixed trained policies. For each seed, the evaluation results were first summarized into run-level performance metrics. The five run-level values, rather than individual packet records, were then used as the statistical samples (n=5).

The reported 95% confidence intervals characterize evaluation-seed variability conditional on the trained policies. Two-sided paired *t*-tests were performed using the five matched run-level differences, with Holm–Bonferroni correction applied across the four evaluated delay metrics.

These statistical results do not account for variability due to independent policy training and should not be interpreted as evidence of training-seed robustness.

**Risk-aware baseline.** PRIMAL-CVaR [17] was included as a risk-aware baseline. Its implementation employed a shared discrete actor, twin reward critics, and an implicit quantile network (IQN) cost critic. The networks used two hidden layers with 256 units each, while the IQN employed 64 quantile samples and an embedding dimension of 64.

The CVaR risk level was set to 0.4, corresponding to the upper 40% of the estimated cost distribution. The instantaneous cost was defined as the queueing delay normalized by the delay normalization factor. The cost-limit parameter was set to 8 and converted into a normalized discounted constraint target using the packet TTL and cost discount factor.

The reward and cost discount factors were both 0.99, and the learning rate for the actor, critics, and Lagrange multiplier was $10^{-4}$. The batch size and soft-update coefficient were 2048 and 0.05, respectively.

PRIMAL-CVaR was evaluated under the same network and traffic settings and matched evaluation seeds as MASAC-EVaR. The two methods retained their respective reward formulations, so the comparison concerns their complete routing implementations.

During preliminary training, cost-limit settings of 8 and 10 were considered. The setting of 8 was selected for the reported comparative experiments, whereas the setting of 10 was not included in the formal five-seed evaluation.

### 5.2. Routing Performance Analysis

#### 5.2.1. Comparison of Routing Performance Metrics

The performance of six routing methods, namely SPF, ECMP, MADQN, MASAC, PRIMAL-CVaR, and MASAC-EVaR, based on five evaluation runs with different random seeds, is presented in Table 2. MASAC-EVaR achieves a favorable balance across multiple performance metrics, including throughput, transmission reliability, average delay, and tail delay. It attains an end-to-end throughput of 815.0 Mbps and an average hop count of 4.9, representing a 10.9% reduction relative to MASAC. This result indicates that MASAC-EVaR tends to select shorter forwarding paths while simultaneously reducing delay variability.

Under high-load conditions, MASAC-EVaR achieves a packet loss rate of 0.00%. Compared with MASAC [20], it reduces the average end-to-end delay from 54.2 ms to 43.8 ms and the P99 delay from 87.5 ms to 58.7 ms. Moreover, the average and maximum queueing delays decrease from 9.63 ms and 27.99 ms to 1.98 ms and 12.05 ms, respectively, corresponding to reductions of 79.4% and 57.0%.

Compared with the risk-aware PRIMAL-CVaR baseline, MASAC-EVaR further reduces the average end-to-end delay from 45.3 ms to 43.8 ms and the P99 delay from 70.6 ms to 58.7 ms, corresponding to reductions of approximately 3.3% and 16.9%, respectively. The average queueing delay is also reduced from 3.4 ms to 2.0 ms, while the maximum queueing delay decreases from 29.5 ms to 12.1 ms. Both methods achieve the same average hop count of 4.9, whereas MASAC-EVaR reduces the maximum hop count from 9 to 7. These results show that MASAC-EVaR retains advantages in tail-delay suppression and queue-congestion mitigation even when compared with a dedicated risk-aware routing baseline.

To place these results in the context of recent topology-aware routing research, we further consider the average-performance metrics reported for DGA-IES. Relative to its own benchmarks, DGA-IES reduces the average end-to-end delay by 10.3–58.1% and packet loss by 3.8–20.0%, while improving throughput by 11.1–57.0% [11]. Under the present experimental setting, MASAC-EVaR reduces the mean end-to-end delay by 19.2% relative to MASAC while maintaining zero packet loss and comparable throughput. Its additional benefits are reflected in the risk-sensitive metrics, with reductions of 32.9%, 79.4%, and 57.0% in the end-to-end-delay P99, mean queueing delay, and maximum queueing delay, respectively. Because the two studies adopt different experimental settings and baselines, this cross-study comparison is intended to provide context rather than a direct performance ranking.

These results demonstrate that the low-return action risk regularization term effectively discourages low-return, congestion-prone actions, thereby alleviating queue congestion and reducing delay variability.

In comparison, MADQN [12] and MASAC exhibit substantially higher queueing and tail delays, while PRIMAL-CVaR achieves lower delays than conventional MASAC but still shows higher P99 and maximum queueing delays than MASAC-EVaR.

The seed-level statistical comparison between MASAC and MASAC-EVaR is presented in Table 3.

As shown in Table 3, MASAC-EVaR achieves lower delays than MASAC across the five matched evaluation seeds. All four paired differences are negative, and their reported 95% confidence intervals exclude zero. The corresponding paired tests also yield Holm-adjusted *p*-values below 0.001.

These results provide evidence of consistent performance differences between the two fixed trained policies under the evaluated stochastic conditions.

#### 5.2.2. End-to-End Latency Decomposition Analysis

**Figure 4:**
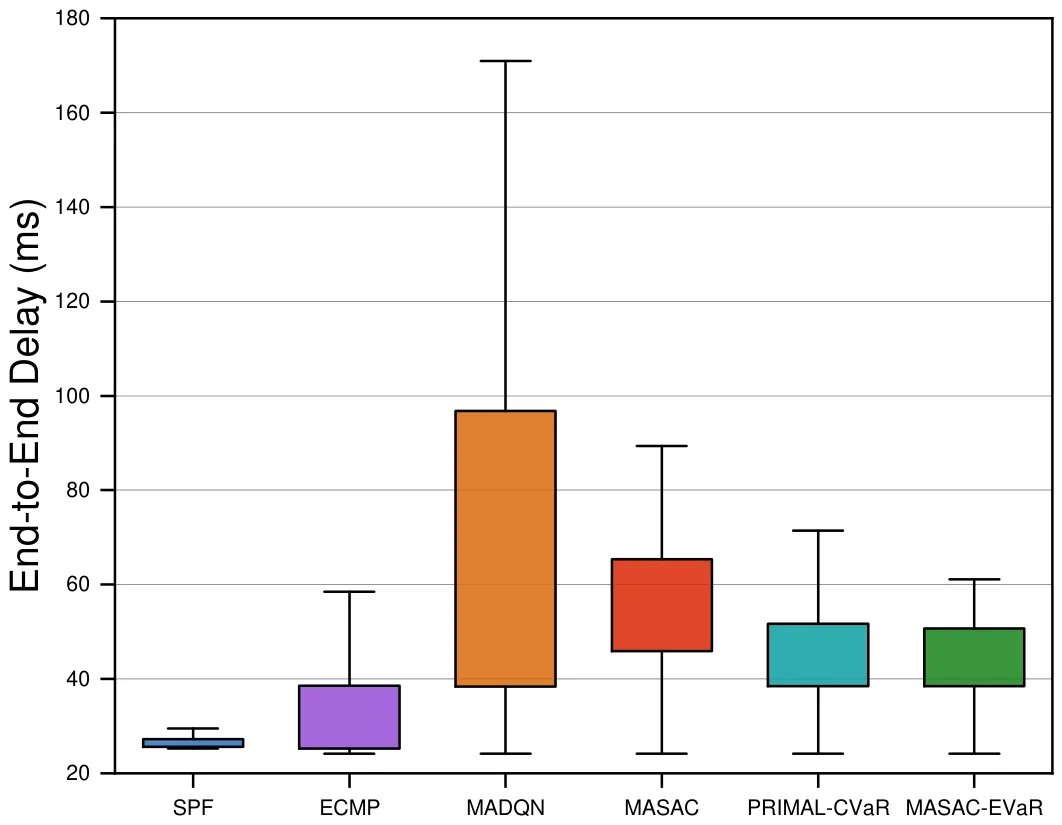
End-to-end delay distribution.

The distributions of end-to-end latency under different routing strategies are illustrated in Figure 4. MADQN exhibits the widest latency distribution, with its upper whisker extending to approximately 170 ms, indicating pronounced extreme tail latency. Although MASAC reduces latency variability, its median latency and dispersion remain relatively high.

PRIMAL-CVaR further reduces the latency dispersion relative to conventional MASAC, with its upper whisker decreasing from approximately 91 ms to 71 ms. MASAC-EVaR and PRIMAL-CVaR exhibit similar median delays of approximately 45.8 ms; however, MASAC-EVaR has a narrower interquartile range and a shorter upper whisker of approximately 62 ms. This comparison indicates that MASAC-EVaR more effectively suppresses extreme-latency events.

**Figure 5:**
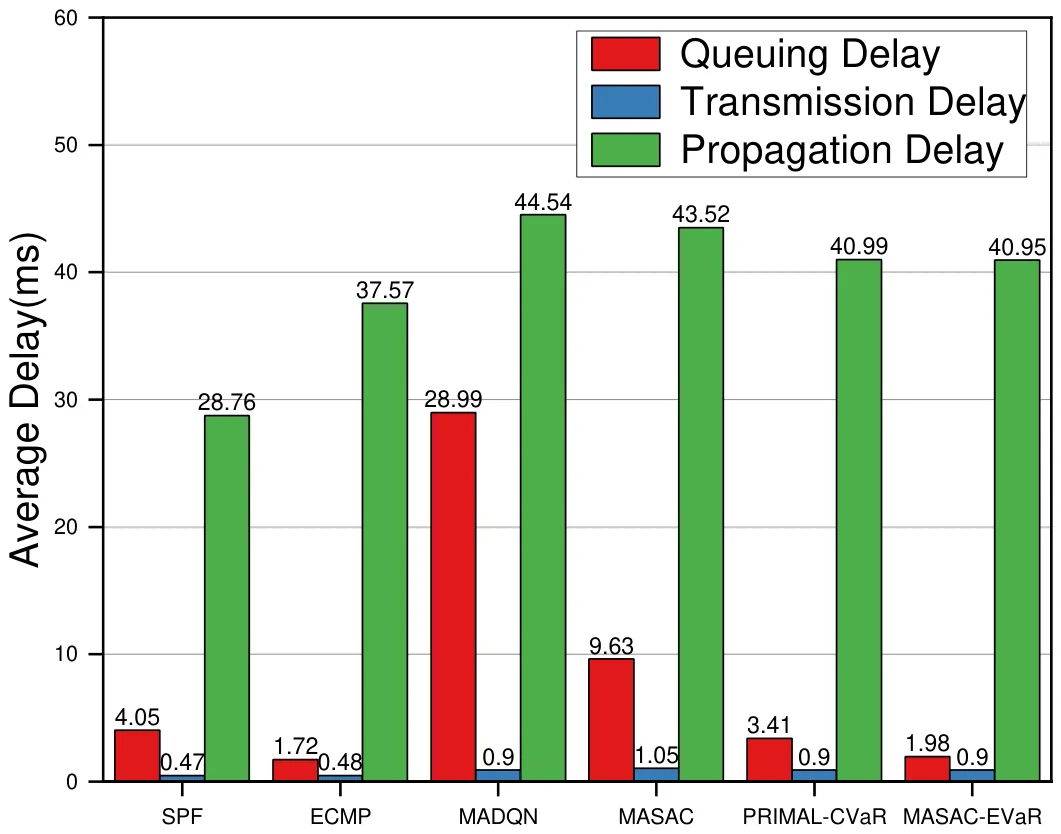
Comparison of end-to-end latency components.

The end-to-end latency is further decomposed into its constituent components in Figure 5. The transmission delay remains low across all methods, while the differences in end-to-end latency are primarily attributable to queueing and propagation delays. MADQN incurs the highest queueing delay, reaching 28.99 ms. MASAC reduces this value to 9.63 ms, PRIMAL-CVaR further reduces the average queueing delay to 3.41 ms, whereas MASAC-EVaR achieves the lowest value of 1.98 ms, representing a 41.9% reduction relative to PRIMAL-CVaR.

PRIMAL-CVaR and MASAC-EVaR have nearly identical transmission delays of approximately 0.90 ms and comparable propagation delays of 40.99 ms and 40.95 ms, respectively. Therefore, the additional end-to-end delay reduction achieved by MASAC-EVaR over PRIMAL-CVaR is mainly attributable to improved queue-congestion mitigation. Overall, these results demonstrate that MASAC-EVaR effectively reduces both average and tail latency while maintaining efficient route selection.

#### 5.2.3. Queueing Delay Distribution and Tail Risk Analysis

**Figure 6:**
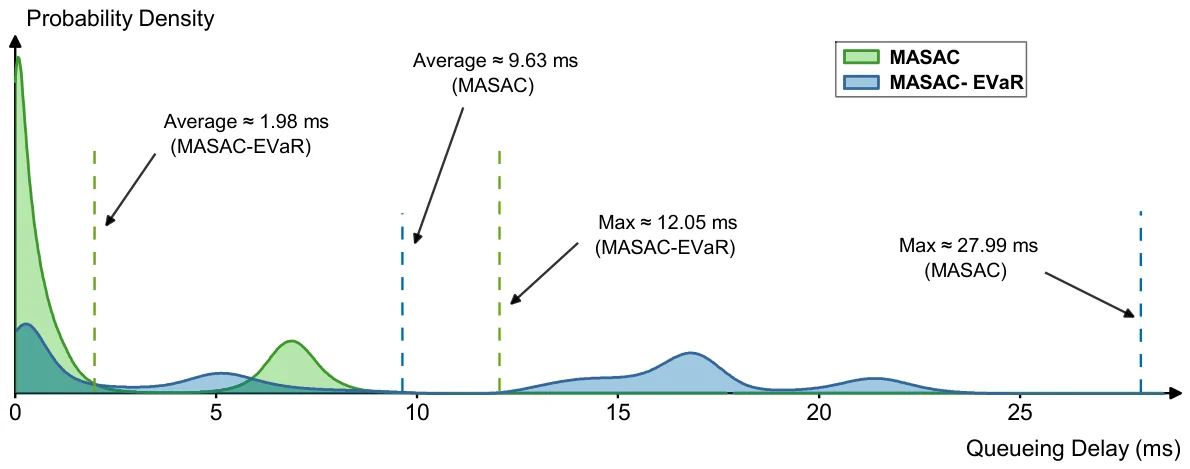
Probability density of queueing delay.

The probability distribution of queueing delay further reveals the differences in tail-delay performance among the routing strategies. Figure 6 compares the probability density functions of queueing delay for MASAC and MASAC-EVaR. Compared with MASAC, the queueing-delay distribution of MASAC-EVaR shifts markedly toward lower delays. Specifically, the average queueing delay decreases from 9.63 ms to 1.98 ms, while the maximum queueing delay decreases from 27.99 ms to 12.05 ms, corresponding to reductions of 79.4% and 57.0%, respectively. These results demonstrate that the low-return action risk regularization term effectively reduces the occurrence of high-queueing-delay events and suppresses the long-tailed behavior of the delay distribution, thereby improving the algorithm’s congestion-avoidance capability and robustness to delay variations.

### 5.3. Queueing Tail-Risk Performance Under Different Offered Loads

**Figure 7:**
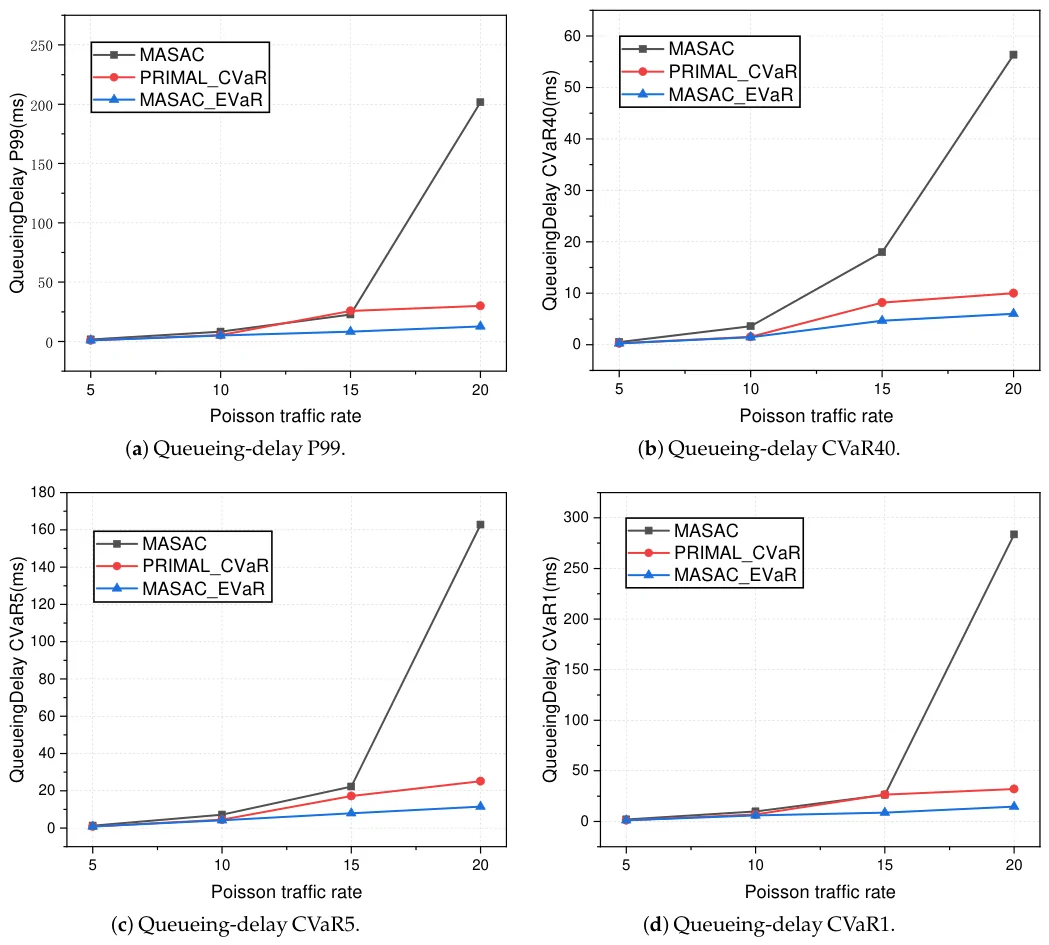
Comparison of queueing-delay tail metrics (P99: 99th percentile; CVaR: conditional value-at-risk) among three learning-based routing methods under different offered loads.

To evaluate the robustness of MASAC-EVaR under different network operating conditions, we conduct additional experiments at packet arrival rates of λ∈{5,10,15,20} packets/ms, corresponding to low, moderate, near-saturation, and highly congested conditions, respectively. Figure 7 compares the queueing-delay P99, CVaR40, CVaR5, and CVaR1. Here, CVaR40, CVaR5, and CVaR1 denote the average queueing delays among the worst 40%, 5%, and 1% of delivered packets, respectively.

Figure 7 focuses on three learning-based routing methods: MASAC, PRIMAL-CVaR, and MASAC-EVaR. This comparison examines their queueing-delay tail behavior under different offered loads. The conventional SPF and ECMP methods are included in the comprehensive performance comparison in Table 2. Their high packet-loss rates at the nominal load make tail-delay comparisons based only on successfully delivered packets less directly comparable.

Under low-load conditions, the three learning-based methods exhibit relatively similar tail-delay performance. As the offered load increases, MASAC-EVaR demonstrates increasingly favorable queueing-delay tail metrics.

As the offered load increases, the advantages of MASAC-EVaR in queueing-delay tail metrics become more pronounced. At λ=15 packets/ms, MASAC-EVaR achieves a queueing-delay P99 of 8.21 ms, compared with 22.64 ms for MASAC and 25.82 ms for PRIMAL-CVaR. Similarly, its CVaR1 is 8.64 ms, compared with 23.257 ms and 26.51 ms, respectively. At λ=20 packets/ms, MASAC-EVaR maintains a queueing-delay P99 of 12.71 ms and a CVaR1 of 14.66 ms, compared with 29.96 ms and 32.05 ms for PRIMAL-CVaR. The CVaR40 and CVaR5 results show similar performance trends.

Overall, MASAC-EVaR achieves favorable queueing-delay tail performance across the evaluated traffic loads, with its relative advantages becoming more evident under heavier traffic conditions.

### 5.4. Sensitivity Analysis of the Low-Return Action Risk Regularization Weight

To investigate the effect of the low-return action risk regularization weight β on system performance, a systematic sensitivity analysis was conducted over the range β∈[0.1,1.0]. In terms of reliability, the packet loss rate remained below 0.02% across all weight settings, indicating that the algorithm is robust to variations in the low-return action risk regularization weight.

**Figure 8:**
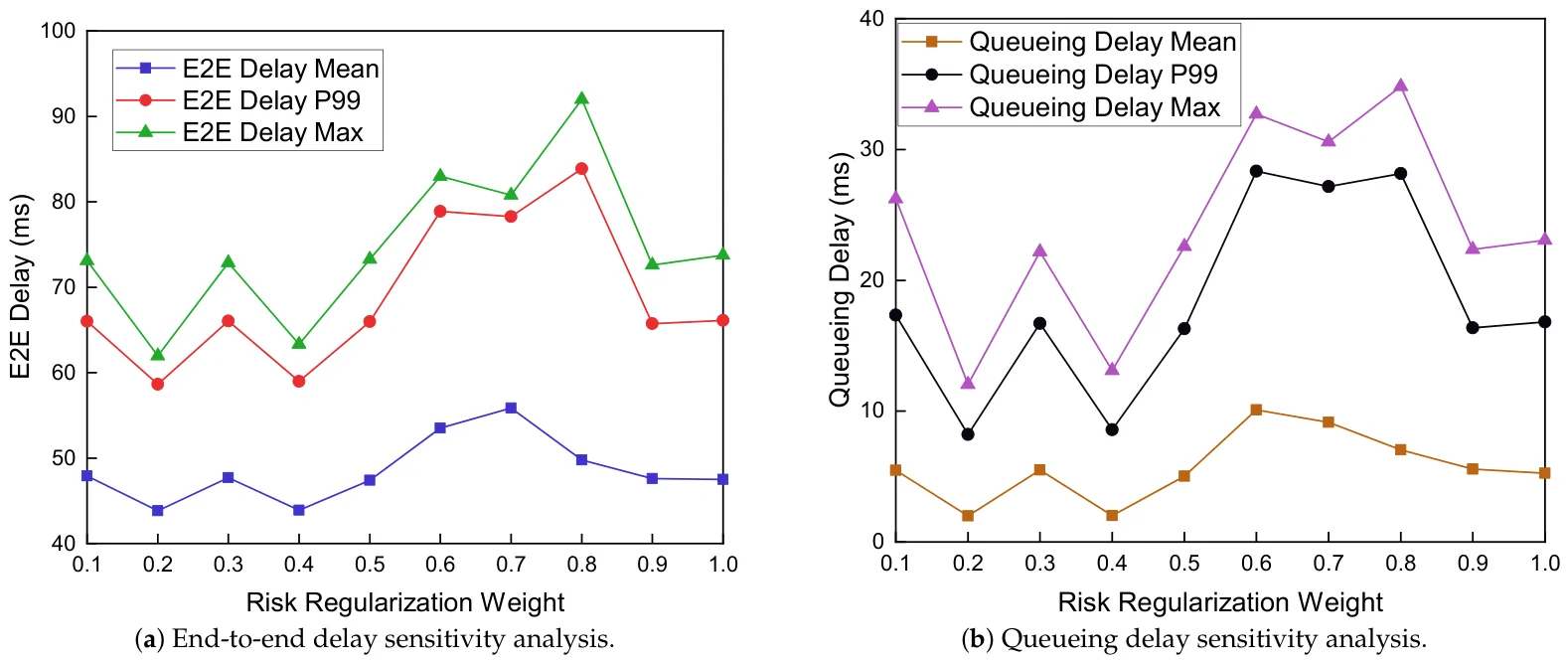
Sensitivity analysis of the risk regularization weight for low-return actions.

The variations in the mean, P99, and maximum end-to-end and queueing delays with β are illustrated in Figure 8. When the risk regularization weight is too small, the resulting risk constraint is insufficient to effectively suppress high-delay events. Conversely, an excessively large weight causes the algorithm to over-penalize low-return actions, which may lead to load imbalance and queue buildup. Considering the mean, P99, and maximum values of both end-to-end and queueing delays, Among the evaluated settings, β=0.2 provided a favorable balance across the considered delay metrics and was therefore adopted in the comparative experiments. Since this selection was based on the same simulation setting, its generalization to independent scenarios remains to be further investigated. These results reveal an inherent trade-off between risk-regularization strength and overall delay and load-balancing performance.

In the present study, the risk-sensitivity coefficient η was set to 0.6 and kept constant throughout the experiments to maintain a consistent regularization configuration. The parameter sensitivity analysis primarily focused on β, which controls the contribution of the entropic regularizer to the actor objective. Keeping η fixed allows the influence of β to be examined without simultaneously varying the risk-sensitivity coefficient. A more comprehensive investigation of different η settings may be conducted in future work to further characterize the parameter sensitivity of the proposed method.

## 6. Conclusions and Future Work

To address the persistent tail-latency problem in risk-neutral reinforcement-learning-based routing methods, this paper proposes an EVaR-inspired low-return action risk-regularized routing algorithm for low-Earth-orbit (LEO) satellite networks. By incorporating a state-level low-return action risk proxy into the MASAC actor loss, the proposed algorithm explicitly limits the probability mass assigned by the policy to low-return actions, thereby jointly optimizing the expected routing return and suppressing the risk associated with low-return actions. Under the evaluated high-load simulation conditions, MASAC-EVaR achieved zero packet loss while reducing the average end-to-end latency, P99 latency, average queueing delay, and maximum queueing delay to 43.8 ms, 58.7 ms, 2.0 ms, and 12.1 ms, respectively. Compared with the baseline algorithms, the proposed method exhibits lower network congestion and extreme-delay events, as well as greater routing stability, enabling efficient and reliable routing in LEO satellite networks under heavy traffic loads.

Future work will further investigate the robustness of the proposed method across independently trained policies and different parameter settings. Additional ablation studies will help clarify the contribution of the entropic regularizer and its relationship with policy optimization. These investigations will further strengthen the generalizability and theoretical understanding of the proposed approach.

Future work may incorporate an explicit delay cost or a dedicated cost critic network. More complex scenarios, including multilayer satellite networks, heterogeneous QoS requirements, and bursty traffic flows [21], may also be investigated to improve the theoretical soundness, generalization capability, and scalability of the proposed algorithm for large-scale deployment. Future work will investigate integrating graph-attention-based topology representation and incremental evolutionary adaptation into the proposed risk-aware routing framework to enhance topology awareness and policy adaptation under dynamic LEO network conditions.

## Figures and Tables

**Table 1:** Simulation parameter settings.

Parameter	Value	Parameter	Value
Number of Satellites	66	Traffic Type	Poisson
Orbital Altitude	780 km	Poisson Rate	15 packets/ms
Orbital Inclination	86.4°	Small Packet Size	16 kbit (20%)
Minimum Elevation Angle	$10^{\circ }$	Normal Packet Size	64 kbit (80%)
Number of Ground Stations	3	TTL	64
GSL Data Rate	1.0 Gbps	Simulation Duration	30 s
ISL Data Rate	0.2 Gbps	Topology Update Interval	100 ms
Batch Size *B*	2048	Replay Buffer Size	100,000
Discount Factor γ	0.99	Learning Rate	$10^{-4}$
Soft-Update Coefficient τ	0.05	Risk-Sensitivity Coefficient η	0.6

**Table 2:** Comprehensive routing performance metrics.

Method	E2E Throughput	Drop Rate	E2E Delay		Queueing Delay		Hops	
			Mean ± Std.	P99	Mean ± Std.	Max	Mean	Max
SPF	4.0 Mbps	99.48%	33.3±17.8 ms	101.3 ms	4.1±9.7 ms	53.4 ms	3.3	8
ECMP	237.7 Mbps	66.25%	39.8±34.9 ms	185.3 ms	1.7±10.4 ms	438.9 ms	4.1	63
MADQN	814.0 Mbps	0.01%	74.4±41.1 ms	154.7 ms	29.0±35.4 ms	123.2 ms	5.0	15
MASAC	814.7 Mbps	**0.00%**	54.2±17.2 ms	87.5 ms	9.6±7.9 ms	28.0 ms	5.5	11
PRIMAL-CVaR	814.8 Mbps	**0.00%**	45.3±11.9 ms	70.6 ms	3.4±5.4 ms	29.5 ms	4.9	9
MASAC-EVaR	**815.0 Mbps**	**0.00%**	43.8±10.1 ms	**58.7 ms**	2.0±2.8 ms	**12.1 ms**	**4.9**	**7**

*Note:* Bold values highlight the results of MASAC-EVaR and the tied zero drop rates of MASAC and PRIMAL-CVaR. The maximum queueing delay in this table is the global maximum across the five pooled evaluation runs. In the seed-level statistical analysis, the corresponding value is instead reported as the mean of the five run-specific maximum queueing delays.

**Table 3:** Seed-level statistical comparison between MASAC and MASAC-EVaR at λ=15 packets/ms.

Metric	MASAC	MASAC-EVaR	Paired Difference	Holm-Adjusted *p*
	Mean [95% CI]	Mean [95% CI]	Mean [95% CI]	
Mean E2E delay (ms)	54.205 [54.172, 54.238]	43.833 [43.817, 43.848]	−10.373 [−10.391, −10.354]	<0.001
E2E-delay P99 (ms)	87.535 [87.520, 87.551]	58.653 [58.622, 58.684]	−28.882 [−28.902, −28.863]	<0.001
Mean queueing delay (ms)	9.633 [9.619, 9.647]	1.983 [1.981, 1.986]	−7.650 [−7.663, −7.637]	<0.001
Maximum queueing delay (ms)	27.537 [27.107, 27.967]	11.169 [10.430, 11.908]	−16.368 [−17.130, −15.606]	<0.001

*Note:* Each learning-based method was trained once and evaluated using five matched evaluation random seeds (n=5). The confidence intervals and statistical tests were calculated from the five run-level summary statistics, rather than individual packet observations. They reflect evaluation variability conditional on the fixed trained policies. The paired difference is defined as MASAC-EVaR minus MASAC; therefore, a negative value indicates a lower delay for MASAC-EVaR. For maximum queueing delay, this table reports the cross-seed mean of the five seed-specific maxima, whereas Table 2 reports the global maximum over the pooled packet records.

## Data Availability

The numerical results supporting the findings of this study are presented in the manuscript. As the simulation framework and related algorithms are being further developed for ongoing and future research, the underlying simulation data, source code, and trained model checkpoints are not publicly available at this stage.

## References

[1] Xu X., Zhao G., Han Z., Cai R., Xu C. (2024). Multi-layer LEO satellite network routing algorithm for 6G communications. Mob. Commun..

[2] Zhu X. (2020). Research on Key Technologies of Routing for Large-Scale LEO Broadband Satellite Networks. Ph.D. Thesis.

[3] Zhang H., Jiang C., Kong H., Lin Z., Han L. (2025). LEO satellite routing strategy based on regional traffic-load splitting. Telecommun. Eng..

[4] Ma W., Xiao S., Zhou Q., Sun W. (2025). Ant-colony-optimization load-balancing routing algorithm for LEO constellations based on segment routing. J. Astronaut..

[5] Dun C., Jin F., Tan S., Qi C. (2022). Dynamic routing algorithm for LEO satellite networks based on load balancing. Radio Eng..

[6] Wang C.W., Du J.N., Wang C., Wang W.D. (2024). Hybrid routing algorithm based on service scheduling in software-defined satellite networks. Acta Electron. Sin..

[7] Dong F., Song J., Zhang Y., Wang Y., Huang T. (2023). DRL-Based Load-Balancing Routing Scheme for 6G Space-Air-Ground Integrated Networks. Remote Sens..

[8] Wei Y., Wang H., Wang W., Li Y., Wang L., Zhao Y. A Deep Reinforcement Learning-Based Service Path Prediction Strategy in Multi-Layer LEO Optical Satellite Networks. Proceedings of the 2025 Asia Communications and Photonics Conference (ACP).

[9] Liang P., Wang X. (2025). State-Incomplete Intelligent Dynamic Multipath Routing Algorithm in LEO Satellite Networks. China Commun..

[10] Ran Y., Ding Y., Chen S., Lei J., Luo J. (2025). Fully-Distributed Dynamic Packet Routing for LEO Satellite Networks: A GNN-Enhanced Multi-Agent Reinforcement Learning Approach. IEEE Trans. Veh. Technol..

[11] Rao Z., Zhu Z., Niyato D., Yao Y., Xu Y., Cheng Y. (2025). Dynamic LEO Satellite Routing Approach Based on Deep Graph Attention and Incremental Evolutionary Reinforcement Learning. IEEE Internet Things J..

[12] Lee C., Kim T., Bang I., Chae S. Multi-Agent Deep Q-Network-Based Handover Optimization for Multi-Beam LEO Satellite Networks. Proceedings of the 2025 Sixteenth International Conference on Ubiquitous and Future Networks (ICUFN).

[13] Liu X., Zhou H., Zhang Z., Gao Q., Ma T. (2025). Multipath Cooperative Routing in Ultradense LEO Satellite Networks: A Deep-Reinforcement-Learning-Based Approach. IEEE Internet Things J..

[14] Han Z., Xu C., Zhao G., Wang S., Cheng K., Yu S. (2023). Time-Varying Topology Model for Dynamic Routing in LEO Satellite Constellation Networks. IEEE Trans. Veh. Technol..

[15] Lu X., Wei W., Fu L., Liu L., Wang D. (2024). Topology-aware multi-service routing in software-defined satellite networks. J. Xidian Univ..

[16] Lyu Y., Hu H., Fan R., Liu Z., An J., Mao S. (2024). Dynamic Routing for Integrated Satellite-Terrestrial Networks: A Constrained Multi-Agent Reinforcement Learning Approach. IEEE J. Sel. Areas Commun..

[17] He K., Vu T.X., He L., Fan L., Chatzinotas S., Ottersten B. (2025). Asynchronous Risk-Aware Multi-Agent Packet Routing for Ultra-Dense LEO Satellite Networks. arXiv.

[18] Haarnoja T., Zhou A., Abbeel P., Levine S. Soft Actor-Critic: Off-Policy Maximum Entropy Deep Reinforcement Learning with a Stochastic Actor. Proceedings of the 35th International Conference on Machine Learning (ICML), Proceedings of Machine Learning Research.

[19] Jia L. (2024). A New Coherent Risk Measure of Entropic Value at Risk for Uncertain Systems. J. Uncertain. Syst..

[20] Zhu J. (2024). Research on Routing Optimization for Low Earth Orbit Satellite Networks Based on Deep Reinforcement Learning. Master’s Thesis.

[21] Li L., Shen G., Zhu C., Li Y., Lei L. Diverse QoS Constrained Routing Algorithm for LEO Satellite Networks Based on Deep Reinforcement Learning. Proceedings of the International Conference on Communication Technology (ICCT).

